# Idiopathic Pulmonary Fibrosis in a Young Adult: A Rare Presentation and Management Challenges

**DOI:** 10.7759/cureus.43010

**Published:** 2023-08-05

**Authors:** Shahzeb Saeed, Norhan Mohammed, Niloufar Maktabijahromi, Chukwuyem Ekhator, Muniba Arshad

**Affiliations:** 1 Internal Medicine, Army Medical College, Islamabad, PAK; 2 Pediatrics, St. George's University School of Medicine, True Blue, GRD; 3 Family Medicine, St. George's University School of Medicine, Brooklyn, USA; 4 Neuro-Oncology, New York Institute of Technology, College of Osteopathic Medicine, Old Westbury, USA; 5 Medicine, Jinnah Hospital Lahore, Lahore, PAK

**Keywords:** pulmonary rehabilitation, progressive respiratory symptoms, chronic interstitial lung disease, idiopathic pulmonary fibrosis (ipf), usual interstitial pneumonia pattern

## Abstract

Idiopathic pulmonary fibrosis (IPF) is a chronic, progressive interstitial lung disease commonly seen in older adults. This case study presents a rare occurrence of IPF in a healthy 26-year-old male. He experienced dyspnea, a dry cough, and fatigue for four months. Tests showed lung function abnormalities and typical pneumonia patterns on imaging, confirming IPF. Treatment included pirfenidone and supportive measures. Early recognition and research are vital for managing IPF in young adults due to limited data.

## Introduction

Idiopathic pulmonary fibrosis (IPF) is a chronic and incapacitating interstitial lung disease characterized by progressive fibrosis of the lung parenchyma, resulting in an irreversible decline of lung function and respiratory failure. Typically affecting individuals over 50 years old, IPF carries a grim prognosis with a median survival of 2-5 years [1]. However, this case study presents an exceptional and uncommon occurrence of IPF in a young adult, emphasizing the difficulties in diagnosing and managing this condition in a younger age group.

IPF is characterized by the progressive replacement of healthy lung tissue with fibrous scar tissue, resulting in impaired gas exchange and respiratory symptoms. While the exact cause is unknown, genetic predisposition, environmental exposures, and abnormal wound-healing responses have been proposed as contributing factors. In older adults, IPF is often associated with a history of smoking, exposure to workplace dust or toxins, and comorbidities such as gastroesophageal reflux disease and connective tissue disorders [2].

The appearance of IPF in young adults is extremely rare, leaving limited data about its natural progression and long-term prognosis in this age group. Young adults with IPF present unique challenges to healthcare providers due to the broad differential diagnosis for interstitial lung diseases in this population, encompassing various autoimmune and connective tissue disorders, environmental exposures, and genetic conditions. Prompt and accurate diagnosis is crucial to initiating suitable management strategies since early intervention may slow disease progression and improve outcomes [3].

Diagnosing IPF in young adults requires a thorough evaluation, including a medical history, physical examination, lab tests, pulmonary function tests, imaging, and sometimes a lung biopsy. Differentiating it from other lung diseases is challenging due to overlapping features, necessitating a collaborative approach involving pulmonologists, radiologists, and pathologists [4].

Managing IPF in young adults adds complexity due to the lack of age-specific evidence-based guidelines. The selection of treatment options, such as antifibrotic therapy, supportive measures, and lung transplantation, must be tailored to individual clinical characteristics, disease severity, and potential risks and benefits. Close monitoring of disease progression, lung function, and quality of life is essential to optimize management and enhance outcomes [5].

This case report seeks to raise awareness about the occurrence of IPF in young adults, stressing the need for a high level of suspicion, early diagnosis, and appropriate management. Further research is warranted to gain a better understanding of the characteristics, causes, prognosis, and optimal management strategies for IPF in young adults, as the existing literature is limited in this population.

## Case presentation

A 26-year-old previously healthy male presented at our clinic with a history of exertional dyspnea, a dry cough, and fatigue lasting four months. He had no significant occupational or environmental exposures, no smoking history, and no family history of lung disease. The symptoms began mildly with breathlessness during physical activity but progressively worsened, limiting his daily activities. On examination, he appeared generally well, but lung auscultation revealed fine bilateral inspiratory crackles, mainly at the lung bases and clubbing of the fingers.

To determine the cause of his symptoms, a comprehensive diagnostic workup was initiated. Laboratory tests, including a complete blood count, liver and renal function tests, autoimmune markers, and serologic tests for connective tissue diseases, were all normal. Serological tests for infectious causes were negative.

**Figure 1:**
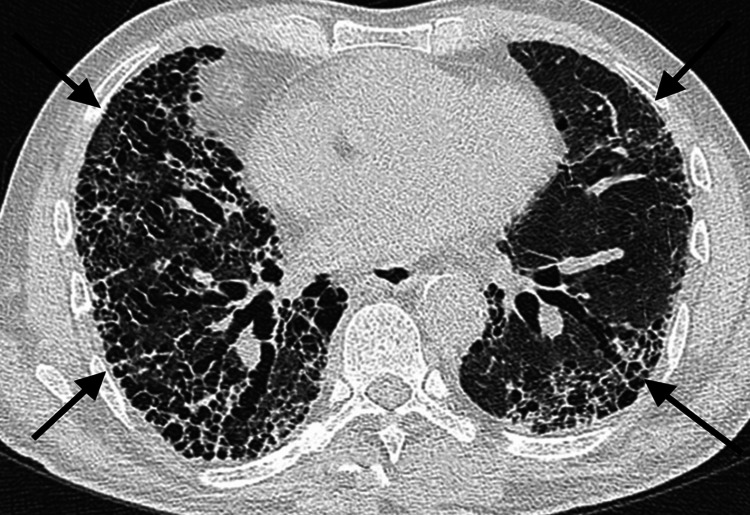
Reticular opacities with honeycombing patterns in an HRCT slice, as marked by arrows.

Pulmonary function tests showed a restrictive pattern with reduced forced vital capacity (FVC) and total lung capacity (TLC), along with significantly reduced diffusion capacity for carbon monoxide (DLCO), indicating impaired gas exchange. A high-resolution computed tomography (HRCT) scan of the chest revealed bilateral reticular opacities with a honeycombing pattern, consistent with the usual interstitial pneumonia (UIP) pattern and supporting a diagnosis of idiopathic pulmonary fibrosis (IPF). The HRCT slice is elaborated in Figure 1.

**Figure 2:**
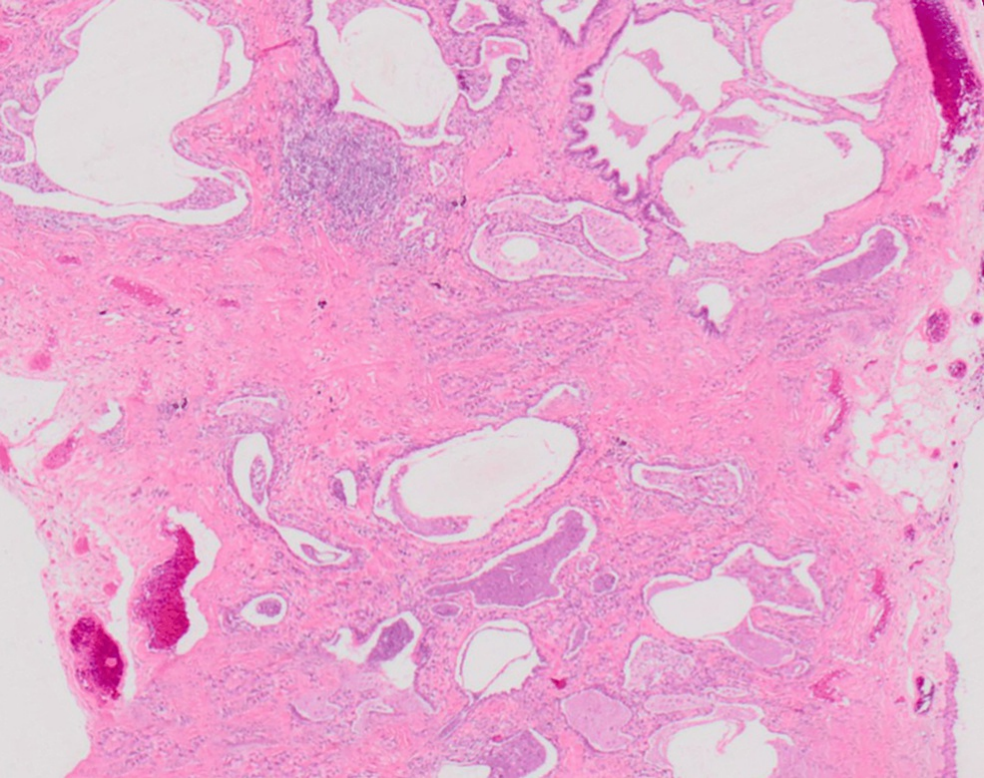
Interstitial fibrosis with dense collagen deposition and fibroblastic foci.

Given the atypical age of presentation and the need to exclude other potential causes of interstitial lung disease, a video-assisted thoracoscopic surgery (VATS) lung biopsy was performed, which confirmed the diagnosis of IPF based on histopathological features of UIP. Histopathological examination of the lung biopsy samples revealed patchy interstitial fibrosis with dense collagen deposition and fibroblastic foci, consistent with the histopathological features of UIP, confirming the diagnosis of idiopathic pulmonary fibrosis. Histological details are elaborated in Figure 2.

The patient was started on pirfenidone, an antifibrotic agent, to slow disease progression and alleviate symptoms. Supportive measures, including pulmonary rehabilitation and supplemental oxygen therapy, were implemented to optimize lung function and improve his quality of life. Regular follow-up visits were scheduled to monitor disease progression and treatment response.

This case emphasizes the rarity of IPF in young adults and highlights the importance of considering it as a potential diagnosis even in this age group. Early and accurate diagnosis of IPF in young adults is crucial for timely intervention and appropriate management to slow disease progression and improve long-term outcomes. Further research is needed to better understand the mechanisms and management strategies specific to IPF in young adults, given the limited data available for this population.

## Discussion

Idiopathic pulmonary fibrosis (IPF) is a complex and challenging disease, particularly when it occurs in young adults, as observed in this case report. While IPF primarily affects older individuals, its occurrence in younger populations is rare, with limited data available on the disease's natural history, progression, and optimal management in this age group [6].

The exact cause of IPF is unknown, but genetics, environmental exposures, and abnormal wound-healing responses are considered contributing factors. In older adults, IPF is commonly associated with smoking, workplace exposures, and comorbidities like gastroesophageal reflux disease and connective tissue disorders. However, in young adults, identifying underlying risk factors is challenging as the disease often occurs without known causative factors [7].

Diagnosing IPF in young adults requires a thorough evaluation and careful exclusion of other potential causes of interstitial lung disease. The differential diagnosis for interstitial lung diseases in this age group is broad and includes various autoimmune and connective tissue disorders, environmental exposures, and genetic conditions. Hence, a multidisciplinary approach involving pulmonologists, radiologists, and pathologists is crucial to reaching an accurate diagnosis [4-5].

In this case, the patient's symptoms of exertional dyspnea, dry cough, and fatigue, along with characteristic physical examination findings, raised suspicion of interstitial lung disease. Pulmonary function tests revealed a restrictive pattern with reduced FVC, TLC, and impaired gas exchange. HRCT imaging of the chest displayed bilateral reticular opacities with honeycombing and traction bronchiectasis, consistent with the UIP pattern, a hallmark of IPF.

To confirm the diagnosis and rule out other causes, a VATS lung biopsy was performed. Histopathological examination revealed typical UIP findings, such as interstitial fibrosis, collagen deposition, and fibroblastic foci. These findings, along with clinical and radiological features, supported the diagnosis of idiopathic pulmonary fibrosis [4-5].

Managing IPF in young adults presents unique challenges due to the limited evidence-based guidelines specific to this age group. Antifibrotic agents, such as pirfenidone and nintedanib, play a key role in IPF management as they have demonstrated efficacy in reducing disease progression and preserving lung function in older adults. However, their effectiveness and safety in young adults need further evaluation, considering potential age-related differences in long-term effects and adverse events.

Supportive measures are essential in managing IPF, regardless of the patient's age. Pulmonary rehabilitation programs, which include exercise training, education, and psychosocial support, can enhance exercise tolerance, alleviate dyspnea, and improve the quality of life. For patients with resting or exercise-induced hypoxemia, supplemental oxygen therapy is recommended to relieve symptoms and enhance oxygenation. Regular monitoring of lung function, symptom assessment, and follow-up visits are crucial to assessing disease progression and treatment response [5].

The long-term prognosis for young adults with IPF is uncertain. In older patients, IPF is associated with a poor prognosis, with a median survival of 2-5 years. However, the disease course and outcomes in young adults may differ, requiring further research for better understanding. Long-term management should prioritize maintaining lung function, improving the quality of life, and considering lung transplantation if the disease progresses rapidly or severely [8].

## Conclusions

This case report sheds light on a rare occurrence of idiopathic pulmonary fibrosis (IPF) in a young adult, underscoring the significance of considering IPF as a potential diagnosis, even in younger individuals. Diagnosing IPF in young adults necessitates a comprehensive evaluation involving clinical, radiological, and histopathological assessments. The management of IPF in this age group should prioritize early intervention, the use of antifibrotic medications, and supportive measures to optimize lung function and enhance the quality of life. Further research is essential to gain a better understanding of the characteristics, prognosis, and optimal management strategies for IPF in young adults.
